# CRISPR-Cas technology a new era in genomic engineering

**DOI:** 10.1016/j.btre.2022.e00731

**Published:** 2022-04-12

**Authors:** Ali Parsaeimehr, Rosemary I. Ebirim, Gulnihal Ozbay

**Affiliations:** Department of Agriculture and Natural Resources, College of Agriculture, Science, and Technology, Delaware State University, Dover, DE 19901, United States of America

**Keywords:** CRISPR-Cas, Gene editing, Agriculture biotechnology, Food industry, Biofuels, Therapeutics

## Abstract

The CRISPR-Cas systems have offered a flexible, easy-to-use platform to precisely modify and control the genomes of organisms in various fields, ranging from agricultural biotechnology to therapeutics. This system is extensively used in the study of infectious, progressive, and life-threatening genetic diseases for the improvement of quality and quantity of major crops and in the development of sustainable methods for the generation of biofuels. As CRISPR-Cas technology continues to evolve, it is becoming more controllable and precise with the addition of molecular regulators, which will provide benefits for everyone and save many lives. Studies on the constant growth of CRISPR technology are important due to its rapid development. In this paper, we present the current applications and progress of CRISPR-Cas genome editing systems in several fields of research, we further highlight the applications of anti-CRISPR molecules to regulate CRISPR-Cas gene editing systems, and we discuss ethical considerations in CRISPR-Cas applications.

## Highlights

• CRISPR-Cas systems offer a flexible and easy-to-use molecular platform to precisely modify and control organisms' genomes in a variety of fields, from agricultural biotechnology to therapeutics.
• With CRISPR technology, crop genomes can be precisely edited in a shorter and more efficient approach compared to traditional breeding or classic mutagenesis.
• CRISPR-Cas system can be used to manage the fermentation process by addressing phage resistance, antimicrobial activity, and genome editing.
• CRISPR-Cas technology has opened up a new era in gene therapy and other therapeutic fields and given hope to thousands of patients with genetic diseases.
• Anti-CRISPR molecules are powerful tools for regulating the CRISPR-Cas systems.

## 1 Introduction

Precise and efficient systems to edit the genome are much desired in biological sciences. Recently introduced molecular tools based on the cluster regularly interspaced short palindromic repeats (CRISPR)-associated nuclease system from the bacterial and archaeal adaptive immune systems are emerging as an effective tool for genome editing in microbial, animal, and plants systems [85, 67]. Characteristically, the CRISPR Cas systems include (a) the gene coding for a CRISPR-associated protein (Cas-protein, b) noncoding RNA sequences, and (c) the repeats dispersed with the short DNA sequences named as protospacers [104]. The Cas proteins require RNA molecules (i.e., CRISPR-RNA and trans-activating RNA in spCas9) for binding and cutting their target on the genome [115]. The protospacers are always linked to 2–6 nucleotides (i.e., 5′-NGG-3′ in spCas9; where “N” can be A, T, C, and G) known as protospacer adjacent motif (PAM). The sequence of the PAM varies among different types of CRISPR systems [38]. Findings demonstrate that, the acquired spacer sequences are greatly identical to each other at the PAMs region, and that this sequence is very essential to the success of the CRISPR system (Table 1). Currently, the classification of CRISPR-Cas systems is based on the specificity of Cas genes, similarity of the CRISPR-Cas sequences, and structure of CRISPR proteins [80]. This categorizes the CRISPR-Cas systems into two classes (I and II), which are further subdivided into six types (type I–VI). Types I, III, and IV are assigned to CRISPR-Cas system class I, whereas types II, V, and VI are assigned to CRISPR-Cas class II [74]. Types I, II, and V of CRISPR-Cas systems are capable of identifying and cleaving the DNA, while type VI can edit RNA, and type III is capable of editing both DNA and RNA [59]. The first documented report [52] of the CRISPR-Cas9 system demonstrated its potential for genome editing and its ability to cleave double-stranded DNA (dsDNA), resulting in double-strand breaks (DSBs). In response, mutations can arise mainly from either non-homologous endjoining (NHEJ) or homology-directed repair (HDR) of DSBs due to errors in the cellular DNA repair machinery at the cleavage site. This precise cleavage of the targeted sequence by RuvC and HNH domains in Cas9 generates DSBs with a blunt end at a location three base pairs upstream of the 3′ edge of the PAM. During the cellular DNA repair process, the NHEJ generates small random insertion or deletions (indels) at the cleavage site, whereas the HDR repair mechanism generates long sequence mutations [[110], [123]]. HDR uses the sister chromatid as a template for its repair and can use external DNA templates for almost any DNA modification required. However, this repair process rarely occurs in nature [29]. On the other hand, NHEJ occurs more rapidly and is more active during the cell cycle (excluding mitosis), whereas HDR is slower and restricted to the S and G2 phases [136].

**Table 1:** The characteristics of the common in use class 2 CRISPR-Cas systems.

Type-subtypes/ nuclease	Size of the protein (aa) / guide spacer length (nt)	Nuclease domains	PAM region	tracrRNA/cut structure/cutting site	Target
II - A/ SpCas9	1368/20	RuvC, HNH	5′ -NGG- 3′ (where “N” can be any nucleotide base)	Yes/blunt end/ ∼3 bp upstream of PAM	dsDNA
II - A/ saCas9	1053/21	RuvC, HNH	5′ -NNGRR(T)- 3′ (where “N” can be any nucleotide base and R is A or G)	Yes/blunt end/ ∼3 bp upstream of PAM	dsDNA
II - B/ FnCas9	1629/21	RuvC, HNH	5′ -NGG- 3′ (where “N” can be any nucleotide base)	Yes/ staggered end/ ∼3 bp upstream of PAM	dsDNA, ssRNA
II - C/ NmCas9	1082/24	RuvC, HNH	5′-NNNNGATT-3′ (where “N” can be any nucleotide base)	No/blunt end/ ∼3 bp upstream of PAM	dsDNA
V - A/ AsCpf1	1307/23	RuvC domain and a putative novel nuclease domain	5′-TTTN-3′ (where “N” can be any nucleotide base)	No/staggered end/ ∼ 19 bp downstream of PAM	dsDNA, ssDNA
V - A/ FnCpf1	1300/21	RuvC	5′-TTN-3′ (where “N” can be any nucleotide base)	No/ staggered end/ ∼ 17 bp downstream of PAM	dsDNA, ssDNA
V - A/ LbCpf1	1228/23	RuvC	5′- TTTV-3′ (where “N” can be A, G, or C bases)	No/ staggered end/ ∼ 18 bp downstream of PAM	dsDNA, ssDNA
V - B/ AacCas 12b (C2c1)	1129/20	RuvC	5′-TTN-3′ (where “N” can be any nucleotide base)	Yes/ staggered end/ ∼ 20 bp upstream of PAM	dsDNA, ssDNA
VI - A/ LshCas13a (C2c2)	1389/28	Helical-1, 2 HEPN	5′-Mononucleotide protospacer-flanking site (PFS) at the 3′-end, having less fit relative to A, U or C	No/-/ ∼ 20-28 bp upstream of PAM. Cleavage activity is dependent on the nucleotide immediately 3’ of the target site	ssRNA

**Note:** The presented Cas systems and their biological origin are as follow, SpCas9 (*Streptococcus pyogenes*), saCas9 (*Staphylococcus aureus*), FnCas9 (*Francisella novicida*), NmCas9 (*Neisseria meningitidis*), AsCpf1 (*Acidaminococcus* sp.), LbCpf1 (*Lachnospiraceae bacterium*), FnCpf1 (*Francisella novicida*), C2c1 (*Alicyclobacillus acidoterrestris*), C2c2 (*Leptotrichia shahii*). dsDNA: Double Stranded DNA, ssDNA: single-stranded DNA, ssRNA: single-stranded RNA.-: No-applicable [[59], [78], [108]].

The CRISPR technology uses programmable RNA molecules for precise editing, unlike other alternative technologies for genome engineering, such as transcription activator-like effector nuclease (TALEN) and the zinc-finger nucleases (ZFNs). Therefore a new protein does not need to be designed and verified for each experiment [34]. The simplicity of the CRISPR-Cas design and its high efficiency made it an attractive tool in the hands of scientists, and as research in CRISPR-Cas science progressed, more CRISPR systems were discovered, introduced, and applied (i.e., Cas12a, Cas13a, and the dead Cas9) [139]. To date, out of 2762 genomes screened in the CRISPR-Cas system database, 1302 bacteria and archaea were found to have a CRISPR system, demonstrating the ubiquity of the CRISPR-Cas system in prokaryotes [133]. Each of these CRISPR-Cas systems has different unique properties based on Cas protein size, PAM recognition sites, and cleavage sites (Table 1). For example, the currently popular spCas9 protein contains 1368 amino acids, and includes a nuclease lube (NUC) and a recognition lobe (REC) [74]. In this review, we discuss the current applications and developments of CRISPR-Cas genome editing technology, highlight the regulatory role of anti-CRISPR molecules in CRISPR-Cas gene editing systems, and finally discuss ethical considerations in the use of CRISPR-Cas gene editing platforms.

## 2 CRISPR for the crop improvement and agricultural biotechnology

Globally, the greatest challenge facing human race is to ensure security for a growing population as the human population is projected to grow to over 10 billion. At the same time, global food productions need to increase by 70% to feed the growing human population [42]. However, in addition to population growth, harsh weather conditions, biotic and abiotic stresses, reductions in agricultural land, water shortage, and pollution pose significant constrains to agriculture and food production [72]. With the introduction of technologies that can help improve crops, production can be increased to some extent. In this regard, the availability of genome sequencing technology and the advancement of CRISPR-Cas systems have opened up potential breeding opportunities for almost every desirable crop. Compared to traditional breeding or classical mutagenesis, CRISPR technology offers a shorter and more efficient approach for precise editing of specific genes in crops [63]. In addition, CRISPR-Cas systems can be delivered directly as ribonucleoprotein (RNP) complexes, containing a Cas protein and the guide RNA. This allows optimization of the Cas protein and guide RNA, increasing transformation efficiency. The use of RNPs reduces off-target effects since they are rapidly degrade and their mode of action is in the treated cells rather than in the regenerated plants [37].

The benefits of CRISPR applications in agriculture have already been confirmed in major crops [98]. In maize (*Zea mays*), CRISPR-Cas9 was used to produce waxy maize hybrids with higher amylopectin. The process was more than one year shorter than classical breeding with marker-assisted selection and backcrossing. The maiz *ZmIPK* gene, responsible for regulating phytic acid synthesis, was also edited by the CRISPR-Cas system, with a higher efficiency (13.1%) compared to the samples obtained with TALENs (9.1%) [70].

In rapeseed (*Brassica napus*)*,* the CRISPR-Cas9 system has also been used to edit two *ALCATRAZ* (*ALC*) homoeologs involved in the development of seed valve margins that contribute to the shattering of mature seeds of tetraploid rapeseed, leading to better resistance to seed loss during mechanical harvest [14]. The CRISPR-Cas9 system was also used to knock out the *CLAVATA3* (CLV3) gene in the *CLV* signaling pathway in the rapeseed, CRISPR-induced CLV mutants produced more leaves and multilocular siliques with significantly more seeds per silique and higher seed weight, which contributed to improved seed production [134]. In barley (*Hordeum vulgare*), SpCas9 was applied to knock out the *HvMORC1* and *HvMORC6a* genes (58.3% mutation efficiency in T0 plants) to study the involvement of these genes in immunity. As a result, infection assays showed that these genes play an essential role barley resistance to biotrophic fungi (*Blumeria graminis*) and necrotrophic fungi (*Fusarium graminearum*) [35].

Cotton is the most important fiber crop and one of the leading agricultural crops in the world. The increased root surface area of the cotton plant (*Gossypium hirsutum*) due to the development of lateral roots, could help it grows faster, which will result in higher fiber yield under drought and low soil fertility conditions. The previous studies show that, the over expression of *OsARG* (rice arginase gene) in cotton has a negative influence on the regulation of the root surface areas. *OsARG* genes were knocked out on both the A- and d-chromosomes of the upland allotetraploid cotton using CRISPR-Cas9 genome editing technology. As a result, CRISPR-Cas9 cotton mutants formed more lateral roots than wild type cotton, which could lead to a higher biomass [128]. The CRISPR-Cas system has also been reported to be effective in genome editing of grape (*Vitis vinifera*, Thompson Seedless cultivar) suspension culture using an *Agrobacterium*-mediated transformation technique to edit the transcription factor *VvWRKY52* gene on chromosome 16, which has been shown to play a role in biotic stress. Analysis of the grape lines edited with CRISPR-Cas revealed that fifteen lines had biallelic mutations, while seven were heterozygous, and sequencing of potential off-target sites revealed no off-target occurrences. Consequently, infection assays with CRISPR-Cas *VVWRKY52* edited grape showed increased resistance to *Botrytis cinerea* contamination [68] *.*

Unsaturated fatty acids play a key role in the structure and function of cell membranes in response to various environmental situations. Fatty acid desaturase 2 (*FAD2*) is the key enzyme responsible for the conversion of oleic acid to linoleic acid. The CRISPR-Cas system was used to knock out the *OsFAD2–1* gene to genetically generate fatty acid profile with high oleic and low linoleic acid content in rice (*Oryza sativa*). As a result, the content of oleic acid increased by more than 200% compared to wild type, and interestingly, linoleic acid, a catabolite of oleic acid by *FAD2*, was reduced to undetectable levels [1].

Resistant starches are starch molecules that resist digestion, which function like dietary fiber and cannot be digested by amylases (resistant starches have attracted significant attention since they are beneficial in preventing various diseases such as diabetes). The rice starch is low in resistant starch and has a high glycemic index. In the Japonica rice (cultivar TNG82), the CRISPR-Cas9 editing of the OsSBEIIb gene resulted in increased resistant starch production and decreased reducing sugar production, as a result, the glycemic index in homozygous and heterozygous CRISPR-Cas mutant rice endosperms was reduced by 28% and 11%, respectively [122].

Tomato (*Solanum lycopersicum*) is one of the most cultivated and consumed plants, and one of the most important dietary sources, which may help reduce the risk of heart disease and cancer. Tomato also contains a high concentration of γ-Aminobutyric acid (GABA) that is known as a non-proteinogenic amino acid with stimulatory roles in central nervous system control of arterial blood pressure. The Glutamate decarboxylase has been identified as a regulatory enzyme in GABA biosynthesis [96]. The CRISPR-Cas system was used to edit a C-terminal auto-inhibitory domain in Glutamate decarboxylase, aiming to increase the production of GABA. As a result, a high-GABA accumulation tomato (125 mg/100 g FW) was produced, and up-regulating the GABA levels can potentially improve the blood pressure-lowering function of tomato fruit [88]. The long-shelf-life is an important factor for the quality of flashy fruits (i.e., tomato and blueberries), since it affects the product's marketability and consumption. In tomatoes, the editing of the ALC gene by the *Agrobacterium*-mediated CRISPR-Cas system resulted in a delay in color change and early ripening, without affecting fruit maturing and harvest-time [137, 69].

A successful application of the CRISPR-Cas system in plants requires an efficient and robust method of delivery into the cells (Table 2). In this regard, biolistic particle delivery (gene gun) and *Agrobacterium*-mediated delivery are the most commonly used CRISPR-Cas delivery methods; however, they have disadvantages, such as limited efficiency, genome sequence damage, foreign DNA integration, and lengthy tissue culture procedures [28]. Alternatively, new delivery techniques, such as virus-mediated gene editing, *de novo* induction of meristem, and haploid-inducer mediated genome editing could be used to improve the CRISPR-Cas editing efficiency and reduce the length of tissue culture (Table 2). In the *de novo* meristem induction the developmental regulators (DRs) and the CRISPR-Cas system will be delivered to the somatic cells, which in response will induce the meristem generated by the genome-edited shot. This system has been applied in *Nicotiana benthamiana* using two strategies: (a) a Fast-TrACC system that is ideal for the identification of the optimal combinations of DRs for meristem induction, and (b) a direct *ex vitro* induction of genome-edited shot, that excludes the need for aseptic techniques and tissue culture [77]. Using plant viruses as a delivery system for CRISPR-Cas constructs is also documented as a promising approach to exclude the long tissue culture procedures since the viruses replicate and travel around their host cells [33]. Currently, both DNA and RNA viruses have been confirmed to be effective in the delivery of CRISPR-Cas constructs. Among the plant virus family, Gemini viruses (GE) are considered as one of the ideal choices for the delivery of CRISPR-Cas constructs, since GE is potent to infect a broad spectrum of plants and they can be used as efficient vectors for the delivery of CRISPR-Cas to multiple hosts [119]. Moreover, GE replicates high amounts of replicons inside their host cells, which in return produce a higher ratio of CRISPR-Cas induced mutations in their hosts [91].

**Table 2:** Applied CRISPR-Cas systems and their delivery methods in agricultural biotechnology.

Edited Plants	De-M	Applied CRISPR-Cas system	Edited genes	Reference
Physcomitrella patens	PEG	Cas9 system using pAct to drive SpCas9, and pU6 to drive sgRNA	PpAPT	[24]
Triticum aestivum	PEG	CRISPR-Cas9 RNP complexes	GW2-B, PinB-D, and ASN2-A	[16]
Glycine max	PEG/Ag-M	Codon optimized Cas9 system, using CaMV35 to drive pCas9, and AtU6 / GmU6 to drive sgRNA	Glyma08g02290, Glyma12g37050,and Glyma06g14180	[111]
Oryza sativua	Ag-M	Plant codon optimized Cas9 system using CaMV35 / pUbi to drive Cas9, and OsU3 and OsU6 to drive sgRNA	OsSWEET14	[138]
Musa spp.	PEG	Cas9 system using pUbi to drive Cas9 / LbCpf1, and OsU3 to drive guide RNA. CRISPR-CAS9 RNP complexes	PDS	[131]
Vitis vinifera	Ag-M	Cas9 system using CaMV35 to drive Cas9, and AtU3 / U6 to drive sgRNA	VvMLO3, and VvMLO4	Wan et al., 2020 [120]
glycine max	Ag-M	Codon optimized Cas9 system using enhanced CaMV35 to drive Cas9, and GmU3 / U6 to drive sgRNA	FAD2–2	[3]
Zea mays	Ag-M	Maize codon optimized Cas9 system with a PTG cassette, using Ubi to drive Cas9, and U6 to drive gRNA scaffold	MADS, MYBR, and AP2	[94]
Hordeum vulgare	Ag-M	Cas9 system with a PTG cassette, using pZmUbi-1 to drive Cas9, and TaU6 to drive the gRNA scaffold	HvCKX1, HvCKX3, and Nud	Gasparis et al., 2018 [36]
Solanum tuberosum	Ag-M	Cas9 system using 2 × CaMV35 to drive Cas9, and U6/U3 to drive sgRNA	PDS	[5]
Oryza sativua	Ag-M	Multiplex genome editing Cas9 system, using CaMV35 / Ubi to drive Cas9, and U6/U3 to drive sgRNA	OsAAP3	[75]
Oryza sativua	Pa-bom	Cas9 system using 2 × CaMV35 to drive Cas9, and OsU3 / TaU6 to drive the sgRNA	OsPDS, OsDEP, and TaLOX2	[102]
Oryza sativua	Pa-bom	CRISPR-SpCas9 RNP complexes	OsPDS1	[4]
Nicotiana benthamiana	Vir	Cas9 system using pSYNV vector with tgtRNA/gRNA expression cassette	PDS, RDR6, and SGS3	[76]

Note: De-M: Delivery method, PEG: Polyethylene glycol, Ag-M: Agrobacterium-mediated, Pa-bom: Particle bombardment, Vir: Viruses, pU6: Physcomitrella patens u6 promoter, pUbi: Z. mays ubiquitin promoter, OsU3: O. sativa U3 promoter, OsU6: O. sativa U6 promoter, pZmUbi-1: Z. mays polyubiquitin-1 promoter, AtU6: Arabidopsis U6 promoter, GmU6: Glycine max U6 promoter, TaU6: T. aestivum U6 promoter, pCas9: Streptococcus pyogenes, OsCas9: O. sativa codon-optimized Streptococcus pyogenes, PTG: polycistronic tRNA-gRNA cassette.SYNV: Sonchus yellow net rhabdovirus, tgtRNA: tRNA-gRNA-tRNA.

Another new approach, the haploid-inducer mediated genome editing (IMGE) technology based on in vivo haploid induction, is becoming a valuable technique to improve the efficiency and reduce the length of breeding procedures in maize [48]. Wang et al., [121] reported a combination of *Agrobacterium*-mediate delivery of CRISPR-Cas constructs and IMEG method and were able to edit the *ZmLG1* gene which is responsible for the leaf angle in maize. The leaf angle in the CRISPR-Cas edited mutants has significantly decreased due to the lack of ligules and auricles.

## 3 CRISPR-Cas systems applied in food industry

**Fig. 1:**
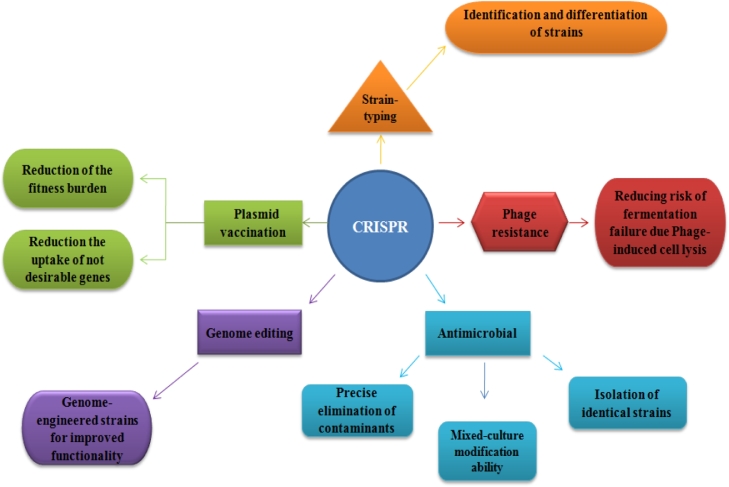
Scheme on applications of CRISPR/Cas-based technology to manage bacteria in food science. Note: Strain typing: studies the microbial evolution, analysis of population-level genotypes in diverse environmental sample types, and strain diversity and relatedness. Phage resistance: Phage-related infections of starter cultures constitute one of the biggest reasons for fermentation failure. Antimicrobial: Biotechnologists can apply CRISPR-Cas technology to eradicate undesirable microbes from production systems by targeting particular populations of bacteria. Phage resistance: The CRISPR-Cas system can be used to target genomic factors that promote phage replication.

Industrial microbes, such as starter cultures and probiotic strains (i.e., *Lactobacillus acidophilus, Lactobacillus casei*, and *Bifidobacterium bifidum*), are ideal targets for CRISPR-Cas genome editing systems due to their important impacts on the food supply chain and their biological functions from the food fermentation to human health [17] **(**Fig. 1**)**. In food processing, these microbes play several critical roles, including preserving food through ribosomally synthesized antimicrobial peptides (bacteriocins), producing hydrogen peroxide, enhancing nutrition of food, and enhancing the organoleptic qualities of food [116]. Studies show, in the starter culture and probiotics the microorganisms that produce lactic acid have a higher amount of CRISPR-Cas as a part of their adaptive immune system, with loci appearing at 62.9 and 77% in lactobacilli and bifidobacteria genomes, respectively [46]. CRISPR-Cas can be a valuable system to manage the fermentation process with applications in phage resistance, antimicrobial activity, and genome editing (Fig. 1). In this regard, the CRISPR-Cas system has been applied to improve probiotic characteristics, such as survival rate through the gastrointestinal passage, host colonization, acid and bile resistance, and uptake and catabolism of non-digestible dietary oligosaccharides [[15], [46]]. The CRISPR-Cas system (SpyCas9^D10A^ nickase (Cas9n) was used to engineer the L. *acidophilus* by the pLbCas9N vector harboring cas9n under the regulation of a Lactobacillus promoter. This introduced CRISPR-Cas system was able to generate chromosomal deletion (300 bp - 1.9 kb) with a mutation rate of 35–100% at different loci. Furthermore, the introduced pLbCas9N system revealed adaptability in *Lactobacillus gasseri* ATCC 33,323 and *Lactobacillus paracasei* Lpc-37, demonstrating the effectiveness of this system in phylogenetically distant Lactobacillus species [39].

In another study, a Native Type I-E CRISPR-Cas system with a 5′-AAA-3′ PAM and 61- nucleotides guide RNA was used for genome editing and chromosomal targeting in *Lactobacillus crispatus*. Targeting the exopolysaccharide priming glycosyltransferase gene using this CRISPR-Cas system generated a variety of mutations, including 643-base pair (bp) deletion with an efficiency of 100%, insertion of a stop codon, and single nucleotide alternation [45]. In another study, by Leenay et al., [66] a novel method using CRISPR-Cas9 mediated genome editing was introduced for *Lactiplantibacillus plantarum* based on using a dsDNA on the replicating plasmid rather than a heterologous recombinase and single-stranded oligo (ssODNs). This proposed system successfully added a stop codon in the riboflavin biosynthetic gene (*rib*), silenced the acetate kinase gene (*ackA*), and completely deleted the β-galactosidase subunit open-reading frame (*lacM*) in L. *plantarum.*

Repeated-spacer arrays in conjunction with CRISPR-Cas genes were used to provide more information on the relatedness of various bacterial strains as well as their ecology (i.e., *Lactobacillus casei* and *Lactobacillus paracasei*) [135]. In this regard, the CRISPR-Cas system has been applied to identify certain genotypes of *Streptococcus thermophilus* in heterogeneous populations as a screening tool [100]. This strategy enables the selection for unexpected mutations, and it can be applied to control specific traits, and improve the starter culture phenotype. In *Limosilactobacillus reuteri* a two-step approach using ssDNA and the CRISPR-Cas system was used for targeted codon mutagenesis, improving the number of recoverable recombinants, as well as identification of recombinant cells in bacteria with low recombineering efficiencies. This technology allowed the selection of oligonucleotide-mediated chromosomal deletions up to 1 kb, and it excluded the need for ssDNA recombineering optimization procedures [122].

*Saccharomyces cerevisiae* is one of the ideal microorganisms to produce bio-based chemicals in the food industry because of its safety and convenience. This mesophilic microorganism grows well at 30 °C, however, during the fermentation process; *S. cerevisiae* produces heat which in return will increase the cost of the production system due to maintaining the temperature through cooling in fermenters [141]. The CRISPR-Cas system was used to engineer the growth of *S. cerevisiae* (strain T8–292) to grow well at 39 °C, moreover, this genetically engineered strain has demonstrated higher cell viability at low pH and high ethanol concentrations [84].

## 4 CRISPR-Cas system as a tool for microbial biofuels production

Biofuels are considered a valuable source for replacing fossil fuels, since it reduces the various undesirable impacts of fossil fuels. Biofuels offer the security, sustainability, and regional development, and reduction in greenhouse gas emissions [44]. The CRISPR-Cas system has been used to improve biofuel production. In this regard, CRISPR has been mainly used to inhibit the competitive metabolism pathways, improve the substrate utilization capacity, and reduce the metabolic flux towards enhancement in solvent production [103]. However, the heterologous CRISPR-Cas systems are frequently difficult to introduce into the prokaryote systems, since most of the microbes have native CRISPR-Cas that can potentially disrupt the exogenous CRISPR-Cas systems [80]. The idea of multiplex genome editing by CRISPR-Cas systems has emerged as a key step in this area of science, and in yeast, the initial multiplex genome editing was successfully reported shortly after the first application of CRISPR-Cas9 genome editing [142, 124]. Generally, there are several approaches to designing a CRISPR-Cas system for multiplex genome editing: (1) expression of several guide RNAs in a single guide RNA expression cassette, (2) expression of several of guide RNAs in multiple guide RNA expression cassette, and (3) expression of a single specific guide RNA to target and edit multiple sequences in the genome [83].

Zhang et al., [140] introduced a novel technique for using CRISPR-Cas to edit the *spo0A* and *pyrF*, dehydrogenase (*adhE1* or *adhE2*), and *cat1* genes in *Clostridium tyrobutyricum*. Using this approach and to guarantee a successful transformation with higher editing efficiency, the Type I-B CRISPR-Cas of C. tyrobutyricum (PAM= TCA or TCG at the 5′-end) under control of a lactose inducible promoter for CRISPR array expression (30–38 nt spacers) was used to reduce the toxicity of CRISPR-Cas. This novel multiplex genome editing system successfully deleted the *spo0A* and *pyrF* genes (editing efficiency =100%). As a result, two mutants capable of producing the highest levels of butanol (26.2 g/L) in batch fermentation were obtained by replacing the adhE1 and adhE2 genes with the cat1 gene. In another study on the genome engineering of consortium of *Clostridium cellulovorans* DSM 743B and *Clostridium beijerinckii* NCIMB 8052 by Wen et al., [129] the CRISPR-Cas system was used to knock out the genes encoding acetate kinase (Clocel_1892 and Clocel_3674) which in return pulled the carbon flux trigger towards butyrate production. Wen's team also used the CRISPR interference (A programmable CRISRP system for gene suppression) to boost ethanol production by suppressing the putative hydrogenase gene (*Clocel_2243*). Moreover, to improve the solvent production in *C. beijerinckii* the genes *ctfAB, cbei_3833/3834,* and *xylR, cbei_2385,* and *xylT, cbei_0109* responsible for the organic acids re-assimilation and pentose utilization, were edited using the introduced CRISPR-Cas system. As a result, the genome engineered *Clostridia* was able to decompose 83.2 g/L of deshelled corn cobs and produced 22.1 g/L of solvents including acetone (4.25 g/L), ethanol (6.37 g/L), and butanol (11.5 g/L).

In view of the accessibility of substantial genomic and metabolic data, *Escherichia coli* is considered as one of the most important options for the production of biofuels. The CRISPR and CRISPRi (clustered regularly interspaced short palindromic repeats interference) were used for the production of 1, 4-butanediol (1, 4-BDO) through metabolic engineering of *E. coli* via an artificial pathway that was regulated by six genes (*cat1, cat2, bdh, bld, sucD*, and *4hbd*). The authors claimed that, the CRISPR-Cas engineered *E. coli* was able to produce 0.9 g/L of 1, 4-BDO in 48 h, and at the next step by using a CRISPRi system the production of 1, 4-BDO improved to 1.8 g/L (100% increase) in genome engineered *E. coli* through reduction of gamma-butyrolactone and succinate in the 1, 4-BDO pathway system [130].

In genome editing studies, the key genes, such as phosphoenolpyruvate carboxylase (PEPC) are of interest as they play a major role in cellular metabolism. Using CRISPRi system, Kao and Ng [54] edited *PEPC1* gene in *Chlamydomonas reinhardtii,* and they observed that, *CrPEPC1* mutants had higher lipid concentration and biomass, despite low chlorophyll content. The results could be explained by the role *PEPC* plays in the synthesis of four-carbon oxaloacetate from phosphoenolpyruvate that is then incorporated into the tricarboxylic acid cycle for protein synthesis. Sharing the same molecule from the glycolysis pathway the acetyl-CoA carboxylase forms the malonyl-CoA (a key molecule in the regulation of fatty acids metabolism) through catalyzing the carboxylation of acetyl-CoA [90]. Acetyl-CoA with key roles in the production of fuels and chemicals (i.e., n-butanol, fatty acids, alkane, polyhydroxybutyrate, acetone, 3- hydroxypropionic acid) is another essential target in gene editing studies, and many scientists have focused on the improvement of acetyl-CoA availability through the metabolism pathways [23]. In this regard, [57] designed and employed a CRISPRi system for multiplex suppression of competing genes (*pta, frdA, ldhA*, and *adhE*) involved in *E. coli* production of by-products (lactate, acetate, succinate, and ethanol) and nicotinamide adenine dinucleotide hydrogen (*NADH*) utilization. By this technique, the n-butanol yield and productivity were up-regulated to 5.4 and 3.2-fold, respectively.

Algae are popular candidate for biofuel production due to their high lipid contents, and as the third generation of biofuel, they have been identified as an economically promising feedstock source for biofuel production. The applications of CRISPR-Cas systems have also been studied by scientists to improve the yields of biofuel production by increasing the algal lipid productivity without affecting the growth of the algal cells [30]. In this regard, the CRISPR-Cas system was used to knock out the *ELT* gene responsible for fatty acid degradation in *Chlamydomonas reinhardtii*. As a result, the genome-edited *C. reinhardtii* mutants showed a higher accumulation in lipid (28% of dried biomass) and an apparent change in fatty acid composition to Oleic acid (C18:1, 27.2% increase) [87]. The diatom *Phaeodactylum tricornutum*, which can be found in brackish and marine waters, has been identified for use in multiple industrial applications, including biofuels and recombinant proteins [20]. Serif et al., [101] established an in vivo non-transgenic genome editing method for *P. tricornutum* using CRISPR-Cas RNP system in conjunction with two endogenous markers, *PtUMPS* and *PtAPT*, which allow long-term nuclease expression in the diatom genome. *Tetraselmis* sp. (Platymonas) is another green alga that has been identified as a source for biomass and lipid production [41]. The carbohydrate synthesis in the metabolism pathway of the *Tetraselmis* sp. was inhibited by targeting the ADP-glucose pyrophosphorylase (*AGP*) gene using CRISPR/Cas-RNP complexes. The results of this study showed that, the lipid content in two *AGP* genome-edited lines was significantly increased by 21.1% and 24.1% when compared to the control under nitrogen starvation. Considering that *AGP* converts α-glucose-1-phosphate into ADP-glucose using the Adenosine triphosphate (ATP) as a substrate in the key starch synthesis pathway, knockout of the *AGP* gene increased glucose-1-phosphate and ATP levels. This resulted in a higher yield of lipid production, since the energy and carbon were conserved [21].

## 5 Insights of CRISPR-Cas systems as a therapeutic

**Fig. 2:**
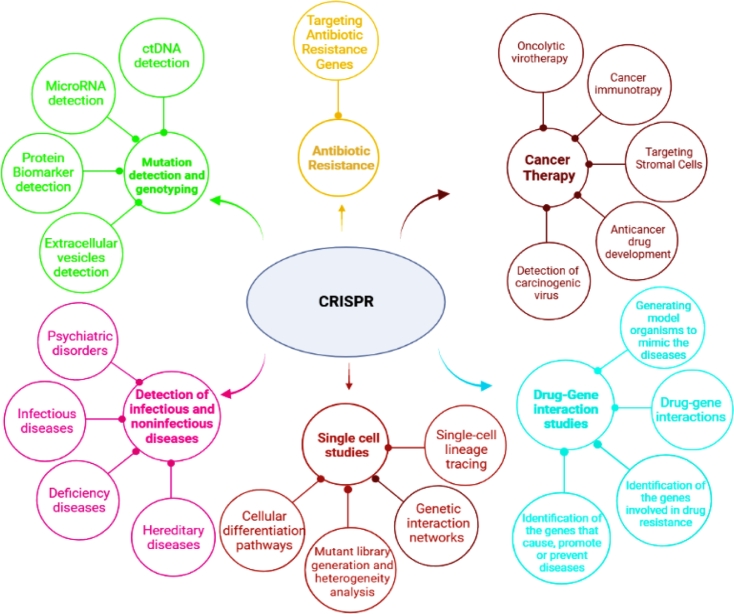
The applications of CRISPR-Cas systems in therapeutics.Note: Gene-editing features of CRISPR-Cas have been used in a variety of therapeutic applications, including cancer diagnosis and therapy, detection of infectious/non-infectious diseases, and genetic disorders. Currently, in the area of therapeutics, CRISPR-Cas is used for experiments, such as testing mutant models, reorganizing the genome, coding-noncoding regions, gene-gene interaction, genetic screens and identifying anticancer immune targets [6, 7, 79].

Gene therapy is a strategy of modification, replacement, or regulation of the affected genes via disruption, correction, or replacement of the affected genes to a level that overturns a diseased phenotypic state [26]. The current progress in CRISPR-Cas technology has opened a new era in gene therapy and other therapeutic fields and provided hope for thousands of patients with genetic diseases (Table 3 and Fig. 2). On 21 June 2016, an advisory committee at the US National Institutes of Health (NIH) accepted a proposal to employ the CRISPR-Cas9 to help cancer therapies that rely on enlisting a patient's T cells, a class of immune cell [27].

**Table 3:** Applied CRISPR-Cas systems and their delivery methods in therapeutic studies.

Targeted cell/model(s)	Linked-disease(s)	Applied CRISPR/Cas system	Delivery system	Edit/Alternation	Reference
HEK 293	Lung adenocarcinoma	Cas9 system using CBh promoter to drive SpCas9, and U6 promoter to drive sgRNA	Plasmid transfection	Chromosomal rearrangement, including CD74-ROS1 translocation and the EML4-ALK and KIF5B-RET inversion events.	[22]
5637 and T24	Bladder cancer	Cas9 system CMV promoter to drive hCas9, and T7 promoter to drive sgRNA	Plasmid transfection	Down regulation of UCA1 gene	[97]
C57BL/Apcfl/fl, KrasG12D/+, and Trp53Δ/Δ mice	Colorectal cancer	Cas9 system using EFS promoter to drive Cas9 and U6 to drive sgRNA	Lentiviral	Suppression of Apc and Trp53 genes in colon epithelial cells	[99]
iPSC and fibroblasts	β-thalassemia	PiggyBac Cas9 expression vector	Plasmid transfection	HBB mutations correction without leaving any genetic footprint in patient-derived iPSC	[132]
CD34+ HSPCs	X-linked chronic granulomatous disease	CRISPR-SpCas9 RNP complexes	Transfection	Regulated expression of cDNA by the endogenous CYBB promoter for functional correction of patient cells	[112]
hiPSC	FTDP-17	dCasRx system using EFS promoter to drive Cas13d, and U6 promoter to drive multiple guides	AAV	Induce exon exclusion to alleviate dysregulation of 4R/3R tau ratios at MAPT exon 10	[58]
Fibroblast cells derived from an HD patient	HD	Specific allele-selective CRISPR/Cas9 system based on PAM-altering SNPs, with an EFS promoter to drive SpCas9, and a U6 promoter for expression of a dual-gRNA cassette	Plasmid transfection	Excise of the spanning promoter region at the mutated HTT gene with complete inactivation of the mutant allele without impacting the normal allele.	[105]

Note: iPSCs: human induced pluripotent stem cells, HBB: Hemoglobin subunit beta, piggyBac: (PB) is a transposon-based vector system. CYBB: Cytochrome B-245 Beta Chain, FTDP-17: Frontotemporal dementia with parkinsonism-17, MAPT: Microtubule Associated Protein Tau, CasRx: Cas system derived from Ruminococcus flavefaciens (XPD3002), hCas9: Human optimized Cas9, UCA1: The lncRNA urothelial carcinoma-associated 1, EFS: EF1 alpha, iPSCs: induced pluripotent stem cells, AAV: Adeno-associated virus, HD: Huntington disease.

CRISPR-Cas systems may also help reduce the leading cause of death in diseases, such as Duchenne muscular dystrophy (DMD), which is a progressive genetic disease that causes muscle degeneration and weakness by altering the protein dystrophin, this protein is responsible for keeping the muscle cells intact [109]. However, CRISPR-Cas delivery systems were reported as an important key element in such therapeutic studies (Table 3). In this regard, Adeno-associated viruses (AAVs) proven effectiveness in delivery of CRISPR-Cas constructs, for example, in the mouse strain C57BL/10ScSn-Dmdmdx/J (mdx) which is a model for DMD studies, the CRISPR-Cas system was successfully delivered by AAVs to the targets at the exon 23 from the Dmd gene [144] The result showed on-target heterogenous genome-editing events at the Dmd locus in all treated mice, and the stability of AAV-CRISPR system for a year. Further studies by Nextera-based sequencing on the cDNA of the genome-edited mice demonstrated, the removal of exon 23 and transcript changes (multi-exon skipping and putative circular RNA formation).

CRISPR-Cas system can be used in clinical settings to target tumors -causing genes. Tumors consist of a population of cells with stem cell-like characteristics (self-renewal, tumor initiation, and progression capacities) that express pluripotency-associated genes (i.e., *NANOG, SOX2, KLFs, Oct-4*, and *c-myc*). These genes are vital transcription factors in embryonic stem cells. Studies have shown that, the expression of NANOG in prostatic adenocarcinoma is related to the proliferation of cancer stem cells [25]. CRISPR-Cas has been used by Kawamura et al., [55] to knock out the NANOG gene, as well as NANOGP8 (Nanog Homeobox Retrogene P8) in DU145 prostate cancer cells. As a result, the in vivo tumorigenic potential (i.e., sphere-forming, anchorage-independent growth, cancer cells migratory, and drug resistance) were significantly reduced in the *NANOG*, and *NANOGP8* knockout cell lines (KHOS and MNNG/HOS). Programmed cell death ligand 1 (PD-L1, also recognized as the cluster of differentiation 274 or B7 homolog 1) is a 40 kDa type 1 transmembrane protein expresses in cancerogenic cells. PD-L1 is another target for studying the effectiveness of CRISPR-Cas systems on tumors. PD-L1 suppresses the T cell-mediated immune response through the PD-1/PD-L1 pathway, and it permits the tumor cells to flee the host's immune system. PD-L1 expression has been used as a predictive biomarker in cancer immunotherapy [53]. PD-L1 gene was knocked down by CRISPR-Cas system in Osteogenic sarcoma (a type of bone cancer) cell lines. The PD-L1 knockdown-cells showed more drug sensitivities for doxorubicin (IC50 = 0.00092 μM) and paclitaxel drugs (IC50 = 0.0020 μM) [71]. At the moment, the PD-1 and PD-L1 cancer immune-therapies need consistent treatment with anti-PD-L1/PD-1 antibodies that may be costly, alternatively CRISPR-Cas systems could be considered as an affordable and easy to use option [79].

CRISPR-Cas systems may also assist in treating disorders, such as transfusion-dependent β-thalassemia (TDT) and sickle cell disease (SCD). TDT and SCD are severe life-threatening monogenic disorders, and both of them are caused by mutations in the hemoglobin β subunit gene, which in return causes ineffective erythropoiesis through an imbalance between the α and β-like globin chains of hemoglobin [117]. BCL11A is identified as a transcriptional factor with a crucial repressor role (silencing) in γ-globin expression and foetal hemoglobin (Hemoglobin F) in erythroid cells [81]. CRISPR-Cas system was used to target the BCL11A in CD34^+^ hematopoietic stem and progenitor cells using electroporation delivery technique. Results showed, the CRISPR-Cas system successfully modified ∼80% of the alleles without any off-target effect [32]. The researchers also reported that, the two cases (one TDT and the other SCD) who have been received genome-edited autologous CD34^+^ cells had higher levels of allelic genome editing in bone marrow and blood. Moreover, the hemoglobin F was recorded higher in both cases, and vaso-occlusive crisis symptoms were eliminated in SCD case [32].

CRISPR-Cas systems have also demonstrated effectiveness in treating autoimmune disorders. In the immune dysregulation, polyendocrinopathy, enteropathy, x-linked (IPEX) syndrome the mutation at the gene forkhead box protein 3 (FOXP3) plays a critical role in maintaining the homeostasis of the immune system [11]. Narrow treatment options (mainly immune-suppressants) are introduced for IPEX, however, these drugs increase the risk of serious side effects linked with toxicity and susceptibility to infections [9]. Currently, the solo curative available option for IPEX is the transplantation of allogeneic hematopoietic stem cells [31]. In this regard, the CRISPR-Cas system was used to edit the IPEX in autologous hematopoietic stem and progenitor cells (HSPCs) and the T cells by inserting a copy of the complementary DNA of the *FOXP3* into the endogenous locus via the HDR pathway, with a targeted integration frequency of 29 ± 8% (using tNGFR marker) [40].

CRISPR–Cas technology has also the potential to cure diseases that have no treatment option available such as the Fragile X syndrome (FXS). FXS is the second most frequent type of intellectual disability (immature neurons, and thin and highly branched dendritic spines) that is caused by the CGG sequence repeats in the promoter region of the FMR1 gene (fragile X mental retardation 1) [89]. CRISPR-Cas systems (Cas9 and Cpf1-RNPs) were used to target the metabotropic glutamate receptor 5 (*mGluR5*) in FMR1 knockout mice using the gold nanoparticles (stereotaxically injection). The results revealed 40 –50% of reduction in expression of *mGluR5* protein and the successful edition (14.6%) in *mGluR5* gene with no significant off-target. Further studies showed that, the tested mice could flee the rhythmic (repetitive) characteristics caused by fragile X syndrome by a single injection dosage of CRISPR/Cas-gold nanoparticles (2.84 µg kg^–1^) [64].

CRISPR-Cas system can be applied to cure life-threatening skin disorders, such as Epidermolysis bullosa (EB). EB is a genetic skin disorder that affects the skin to become extremely fragile. To date, molecular analysis in EB disorder has shown mutations in at least 16 distinct genes (i.e., *KRT5, KRT14, KLHL24, PLEC, DST, LAMA3, ITGB4, COL7A1,* and *LAMB3*) associated with the cellular integrity and adhesion [6]. Bonafont et al. [12] used a dual guide RNA in the CRISPR/Cas-RNP system to edit the exon 80 of the COL7A1 (Collagen alpha-1) gene in the immunodeficient mice (nu/nu, NMRI background) by a direct delivery of CRISPR-Cas RNP using electroporation. As a result, highly efficient editing at Exon 80 was reported (∼85%) without noticeable cellular toxicity. In this study, sustained skin regeneration with a polyclonal population of the edited cells was observed. The analysis of the editing cells by next-generation sequencing analysis showed no off-target effect, confirming the safety of the system.

The increasing demand for accurate detection of related molecules in therapeutics and scientific research has accelerated the progress of advanced molecular diagnostic techniques. Accordingly, the fast, low-cost, and sensitive nucleic acid CRISPR-based detection (i.e., Cas12 and Cas 14 – based detectors, Cas9- Flash, and Cas13-Sherlock) potentially can help in the areas of pathogen detection, genotyping, and disease monitoring (Table 4). The inherent allele specificity of CRISPR is the primary and main reason for its application in molecular diagnostic platforms of infectious and non-infectious diseases [10]. In this regard, Cas13-Sherlock diagnostic platform has been used as a rapid-point of care and mobile diagnostic molecular platform. It is reported that, the Cas13-Sherlock is significantly better than RT-qPCR technique to detect Ebola and Lassa Viruses (100% sensitivity) [7]. In another study, a diagnostic platform based on CRISPR-Cas12a system combined with a fluorescent probe for detection of target amplicons was used for detection of SARS-CoV-2. This technique was reported to be fast (∼50 min) with higher sensitivity, and its results were comparable to those results obtained with a CDC-approved RT-qPCR assay [50]. The CRISPR-Cas12a system has also been used for rapid detection of *Mycobacterium tuberculosis* (Mtb), considerably; the results demonstrated that, the CRISPR- Cas12a Mtb diagnostic platform was very sensitive (near single-copy sensitivity), and requires less sample input, which in return offers a shorter time for analysis in comparison to the other available diagnostic platforms, such as GeneXpert MTB/RIF (Xpert) [2].

**Table 4:** CRISPR-Cas nucleic acid detection approaches and their mechanisms.

Detection platform	Mechanism	Analysis	Sensitivity/portability	Reference
Cas12 –Based Detector	The RPA amplified DNA is used as a template directly. The Cas12 protein guided with the specific gRNA recognizes and targets the specific nucleotide sequences. This causes the collateral cleavage and degradation of the fluorescent reporter (FQ-reporter), and consequently leads to detection of the target presence.	Fluorescence	aM/Yes	[114]
Cas14-Based Detector	Cas14 complex recognizes and binds to ssDNA. The targeted sequence is amplified using the PRA method through specific primers which create resistant T7 exonuclease sequences at the end of dsDNA. By this technique, the unmodified DNA strand will be degraded by T7 exonuclease. Upon target detection and cleavage, the Cas14 collateral activity will result in the degradation of the fluorescent reporter, and subsequently indicates the existence of the target.	Fluorescence	aM/Yes	[49]
Cas9- Flash	The phosphatase-treated genomic DNA or cDNA are used for the Cas9-mediated cleavage of the targeted sequence. The cleaved strands will be ligated to the adapters using specific primers; consequently, the target sequence will be analyzed by sequencing.	Sequencing	aM/No	[95]
Cas13-Sherlock	The PRA-amplified templates from the target are transcribed by T7 in vitro transcription to generate RNA templates. The Cas13 protein guide by specific primers will identify and cleave the target, which resulted in degradation of the FQ-reporter.	Fluorescence	aM-zM/Yes	[56]
(CRISPR)-mediated DNA-FISH detection	The magnetic nano-particles are attached to dCas9, and consequently, the target sequence bind by the dCas9-sgRNA complex will be isolated from the nucleotide pool via magnetic, which in return induces the fluorescence signal through SYBR Green staining.	Fluorescence	aM/Yes	[43]

Note: PRA: recombinase polymerase amplification, FLASH: Finding Low Abundance Sequences by Hybridization, ctPCR detection: The CRISPR-typing PCR, cDNA: Complementary DNA.

## 6 Control of CRISPR/Cas gene editing systems using anti-CRISPR molecules

While currently available technologies enable the activation of CRISPR-Cas systems, new technologies for predictable control and efficient inhibition of this system have yet to be introduced [107]. The increasing applications of CRISPR-Cas systems in agriculture, synthetic biology, and therapeutics have attracted the attention of scientists seeking novel CRISPR-Cas inhibitors as a potential strategy to control gene editing applications [80]. The mode of action of the anti-CRISPR protein is that organisms must constantly develop new mechanisms of resistance to parasites in order not to be threatened with extinction. Phages have evolved a variety of mechanisms to eliminate the CRISPR-Cas system through modification of restriction sites, degradation of restriction modification systems, and expression of specific proteins with the ability to attach and neutralize the CRISPR-Cas defense system [82].

The first reported case of an inhibitor of the CRISPR-Cas system was identified in *Pseudomonas* spp. phages, as the phages were able to contaminate and replicate in *P. aeruginosa* despite having an active I-F CRISPR-Cas (PA14) [13]. Further studies showed that AcrF1, AcrF2, AcrF3, AcrF4, and AcrF5 proteins are responsible for inactivation of the I-F CRISPR-Cas system in *P. aeruginosa* [92]. Currently, thirty anti-CRISPR families (Acr) have been discovered and have been classified into three different types: (1) CRISPR guide RNA inhibitors (i.e., AcrIIA1, AcrIIC2, and AcrVA1, 2) CRISPR/Cas DNA binding blockers (i.e., AcrF1, AcrIIA2, AcrIIC5, and AcrIIC1), and (3) DNA cleavage inhibitors (i.e., AcrIIC3, AcrIIC1, AcrVA5, and AcrIE1) [74].

The practical application of Acrs was investigated by Nakamura et al., [86] using the CRISPR-Cas and CRISPRi systems in different cell types (HiPSC, HEK293T, and *S. cerevisiae*). The results showed that AcrIIA4 has the ability to regulate Cas9 protein activity even when fused to other genes despite the N- or C-terminus. This regulatory capacity was stable for months without evidence of a cytotoxic effect. Bubeck et al. [19] used the protein AcrIIA4 from the prophage *Listeria monocytogenes* III (a Cas9 inhibitor from *Streptococcus pyogenes*, size: 87aa) and the LOV2 photosensor (16.5 kDa) from *Avena sativa* (Oats) to control light-mediated genome editing in human cells (HEK293T and U2OS).

In another study, Hoffmann et al. [47] used a vector containing the CMV promoter-driven AcrIIA4 under the regulation of MicroRNA-122 (miR-122) and MicroRNA-1 (miR-1). miR-122 and miR-1 are RNA genes that play key roles in regulating cholesterol levels in liver and muscle tissues, respectively [127]. Simultaneous expression of AcrIIA4, miR-122, or miR-1 and the CRISPR-Cas system resulted in activation of the engineered system selectively in hepatocytes or cardiomyocytes and inhibition of off-target cells by knockdown of the Acr gene and induction of CRISPR-Cas activity (full-length Cas9, split-Cas9, dCas9-VP64) up to 100-fold. In another study by Jain et al., [51], a chemically inducible CRISPR-Cas system was introduced under the control of a CMV promoter. The system was inducible by activating/inactivating AcrIIA4 as an anti-CRISPR protein by adding trimethoprim (an FDA-approved ligand). This method achieved high specificity of CRISPR-Cas editing with ∼0% off-target activity in mammalian HEK293T cells.

## 7 Ethical considerations for the CRISPR-Cas system

Ethical decisions, especially in biomedicine, are analytically based and involve an assessment of the potential risk-benefit ratio. To find the path to an ethical decision, it is very important and essential to consider the range of possibilities and outcomes, as well as the possible justifications for such a decision. The CRISPR-Cas system is currently the leading technology used in most genetic engineering procedures for targeted editing of a wide range of genomes [126].

CRISPR technology is a promising tool that can be used to improve the treatment of a wide range of diseases, with other promising applications ranging from agriculture to the environment to clinical therapeutics. However, there are ethical and safety concerns worldwide about the use and application of this technology in areas such as germline editing [18, , ].

There are three major concerns about ethical considerations with CRISPR genome engineering technology. These include the possibility of limited on-target editing efficiency, incomplete editing, and the future of the altered organisms: whether they will be affected forever or whether the altered genes will be transmitted to future generations and potentially affect them in unforeseen ways. Other concerns and challenges include human safety and dignity and the risk of exploitation for eugenic purposes, ethical issues such as the risk of unanticipated adverse effects in clinical applications, especially to correct or prevent genetic diseases, and the issue of informed consent [, 106]. The most important ethical concern is the users of the technology, not the technology itself, because these scientists decide how to use CRISPR/Cas9 technology [8]

Another ethical issue of great concern in the application of CRISPR technology is its proposed use to modify human embryos to cure disease or prevent disease in humans before birth. Several highlighted reports have been published that have raised the possibility and risks to whole genome integrity because of subtle mutations that may be a byproduct of off-target CRISPR-Cas actions to which chromosomal alterations may be induced in model embryo systems, leading to potentially serious health problems that may occur during CRISPR application in the human embryo [61, 62, 93].

## 8 Conclusion and future perspectives

CRISPR/Cas systems have provided a flexible, easy-to-use molecular platform for precisely modifying and controlling the genomes of organisms across a wide range of fields, accelerating discoveries in genetics and the treatment and cure of disease through gene therapy. CRISPR-Cas systems have demonstrated their potential to cure life-threatening diseases (e.g., cancer, cystic fibrosis, DMD, TDT, SCD, FXS, and EB) by editing the disease-associated genes with little off-target effects. In agriculture, CRISPR-Cas technologies have demonstrated their potential to increase yield and quality of crop products, enhance crop resistance to drought, herbicides, and insecticides, and extend the shelf life of produce, which in turn improves food safety. Efficient CRISPR-Cas-based genome editing systems have already been developed for genome editing of various microorganisms (e.g., *Clostridium, E. coli, B. subtilis*, and *C. reinhardtii*) to improve biofuel production by reprogramming their metabolic pathways. As CRISPR-Cas technology grows and studies in this field of science progress, the addition of other CRISPR-Cas molecular regulators such as Acrs (i.e., AcrIIA4, AcrF1 and AcrIIC1, AcrVA5) will make the CRISPR-Cas platform more controllable, efficient and precise, which in turn can make a valuable contribution to the safe and practical use of CRISPR-Cas systems in the field of genome editing.

## Conflict of Interest

The authors have no conflicts of interest to declare that are relevant to the content of this article.
